# Drop-Weight Impact Test on U-Shape Concrete Specimens with Statistical and Regression Analyses

**DOI:** 10.3390/ma8095281

**Published:** 2015-09-03

**Authors:** Xue-Chao Zhu, Han Zhu, Hao-Ran Li

**Affiliations:** 1 School of Civil Engineering, Tianjin University, Tianjin 300072, China; E-Mails: 1011205075@tju.edu.cn (X.-C.Z.); haoran078@tju.edu.cn (H.-R.L.); 2 Key Laboratory of Coast Civil Structure Safety, Tianjin University, Ministry of Education, Tianjin 300072, China

**Keywords:** U-shape specimen, concrete, drop-weight test, impact resistance, coefficient of variation, statistical analysis

## Abstract

According to the principle and method of drop-weight impact test, the impact resistance of concrete was measured using self-designed U-shape specimens and a newly designed drop-weight impact test apparatus. A series of drop-weight impact tests were carried out with four different masses of drop hammers (0.875, 0.8, 0.675 and 0.5 kg). The test results show that the impact resistance results fail to follow a normal distribution. As expected, U-shaped specimens can predetermine the location of the cracks very well. It is also easy to record the cracks propagation during the test. The maximum of coefficient of variation in this study is 31.2%; it is lower than the values obtained from the American Concrete Institute (ACI) impact tests in the literature. By regression analysis, the linear relationship between the first-crack and ultimate failure impact resistance is good. It can suggested that a minimum number of specimens is required to reliably measure the properties of the material based on the observed levels of variation.

## 1. Introduction

As it is known, concrete is one of the most widely used building materials in modern architectures. However, in civil engineering, all kinds of concrete structures inevitably encounter dynamic load during the design lifetime, except in the case of static load [1,2]. Dynamic load (such as earthquake load, impact load, explosion load, vehicle load, wave load, wind load, *etc.*) can cause damage to and collapse of buildings, which can lead to casualties and property losses [3]. In addition, the impact resistance is a very important parameter for evaluating the dynamic performance of concrete, and thus it is very necessary to study concrete under impact load.

Several types of impact tests have been used to measure the impact resistance of concrete and similar construction materials [4,5,6,7,8,9]. These can be classified broadly, depending upon the impacting mechanism and parameters monitored during impact, into the following types of tests [10]: (a) weighted pendulum Charpy-type impact test; (b) drop-weight test(single or repeated impact); (c) constant strain-rate test; (d) projectile impact test; (e) split-Hopkinson pressure bar (SHPB) test; (f) explosive test; and (g) instrumented pendulum impact test. However, none of these tests has been declared to be a standard test, at least in part due to the lack of statistical data on the variation of the results, and comparisons between any of the above tests are very difficult. In addition, some of these tests are relatively difficult to perform and require sophisticated equipment. In this regard, ACI Committee 544 [11] has proposed a drop-weight impact test to evaluate the impact resistance of concrete. The test is widely used since it is simple and economical [2,12,13,14,15,16]. However, the results obtained from this test are often noticeably scattered due to the nature of the test and the nonhomogeneous condition of the concrete [2,5,17]. The large variation in impact resistance as determined from this test is reported in the literature [12,13,14,15,16], and the reported coefficient of variation of impact resistance is approximately 50%–60%. The sources of large variations in results obtained from the ACI impact test can be summarized as follows [12]: (a) The cylindrical specimens are used in the test, which allow cracks to occur anywhere and in any direction. This increases the subjectivity of the test and makes the visual identification of the first crack difficult; (b) The criteria for accepted or rejected failure mode is absence, which increases the scatter of the results. Therefore, the variations in mechanical properties should be considered in deciding the minimum number of tests required for measuring material properties.

In order to reduce the large variation in results of the impact test and predetermine the location of the crack, the impact resistance of concrete was measured using self-designed U-shape concrete specimens (Zhang [18], under the supervision of the correspondence author in this paper, explored a number of specimen geometries, and the one with a U-shape appeared a favorable candidate for further study) and a newly designed drop-weight impact test apparatus (based on Marshall instruments and making some modifications) in this paper. The impact test results were subjected to comprehensive statistical analysis and compressive strength tests were also conducted on cubic specimens as a means of quality control. Moreover, by using the regression technique, linear relationships between the first crack and ultimate failure impact resistance in blows were proposed; based on the theory of statistics, the minimum number of specimens in tests of concrete impact resistance was also proposed to keep the error within a certain limit.

## 2. Experimental Program

### 2.1. Materials and Mix Proportions

The P.O42.5 grade ordinary Portland cement (compressive strength is 47.7 MPa at the age of 28 days by the test method of Chinese standard GB/T 17671-1999 [19], which is identical with ISO 679:1989) was used in this study. The chemical composition of the cement is shown in Table 1, as given by the supplier. According to the Chinese Code (GB/T 14684-2011 [20] and GB/T 14685-2011 [21]): (a) the fine aggregate was natural river medium sand with a fineness modulus of 2.50 and maximum size of 5 mm; (b) the coarse aggregate was crushed limestone with sizes of 5–20 mm, and it had an apparent density of 2650 kg/m^3^. Water was tap-water.

**Table 1:** Chemical composition of the cement.

Oxide	SiO_2_	A1_2_O_3_	CaO	MgO	SO_3_	Fe_2_O_3_	Loss on Ignition
Content(%)	22.60	5.03	63.11	1.46	2.24	4.38	1.18

The mix proportions, by weight, were, water:cement:fine aggregate: coarse aggregated = 0.49:1:1.43:2.55, with a *w*/*c* ratio of 0.49 and sand ratio of 36%. The mixture proportions are given in Table 2.

**Table 2:** Mix proportions of concrete (kg/m^3^).

Water	Cement	Coarse Aggregate	Fine Aggregate
215	439	1118	629

### 2.2. Specimen Preparation

**Figure 1:**
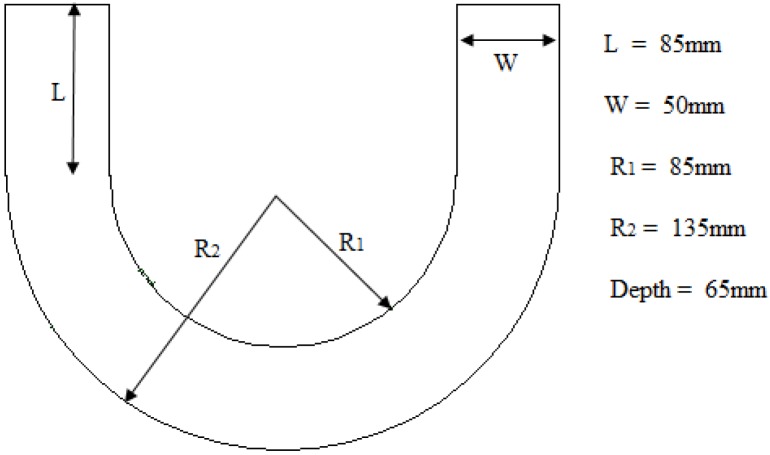
Dimensions of U-shape concrete specimen.

**Figure 2:**
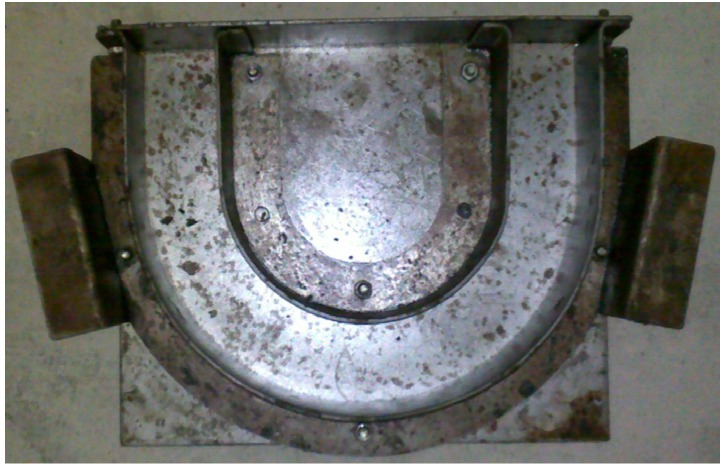
U-shape steel mould.

In order to force cracks to occur in a predefined path during the test, the concrete specimens were made into U-shape, which would cause crack initiation and ensured that the specimens were destroyed in the middle of them. Figure 1 shows the dimensions of the U-shape concrete specimens used in this study. For ensuring accurate dimensions of specimens, steel moulds were made up and used, as shown in Figure 2. Two handles were designed on both sides of each steel mould, in order to conveniently vibrate and carry the specimens in the process of casting concrete. Before casting concrete, the steel moulds were cleaned, and release agent was brushed on the inside surface of steel moulds.

The conventional forced concrete mixer was used. Crushed limestone, sand and cement were respectively weighed by order and mixed in the dry state for 1–2 min, until the materials were mixed homogeneously; then the weighed tap-water was slowly poured into the mixer and the mixture was stirred about 3 min. Workability of the fresh concrete was assessed using the slump test conforming to the Chinese Code (GB/T 50080-2002 [22]). The slump value was 135 mm.

Concrete mixtures were filled into the U-shape steel mould. After casting, the concrete specimens were compacted using a vibrating table. When the paste came out from the mixture, the vibrating table was turned off. The extra concrete was scraped in the end. Spatulas were used to ensure that all specimens had smooth surfaces.

Specimens were cured in natural curing conditions for 24 h. They were then demoulded and numbered. Socket spanners and open spanners were used in the process of demoulding. After that, specimens were immediately placed in the standard curing room, in which temperature was 20 ± 2 °C and the humidity was more than 95% according to the Chinese Code (GB/T 50081-2002 [23]).

Twelve U-shape steel moulds were cast from each batch to prepare specimens for the impact test. Three cubes (100 mm) were prepared from each batch according to the Chinese Code (GB/T 50081-2002) for the compressive strength test. A total of 60 U-shape specimens were cast for the impact test and 15 cubic specimens were cast for the compressive strength test. All specimens were cured in the standard curing room, until testing at the age of 28 days. After 28 days, the specimens were moved out of the standard curing room 4 hours before testing, and then the specimens were cleaned with dusters to dry in the natural environment.

### 2.3. Test Procedures

#### 2.3.1. Drop-Weight Impact Test

**Figure 3:**
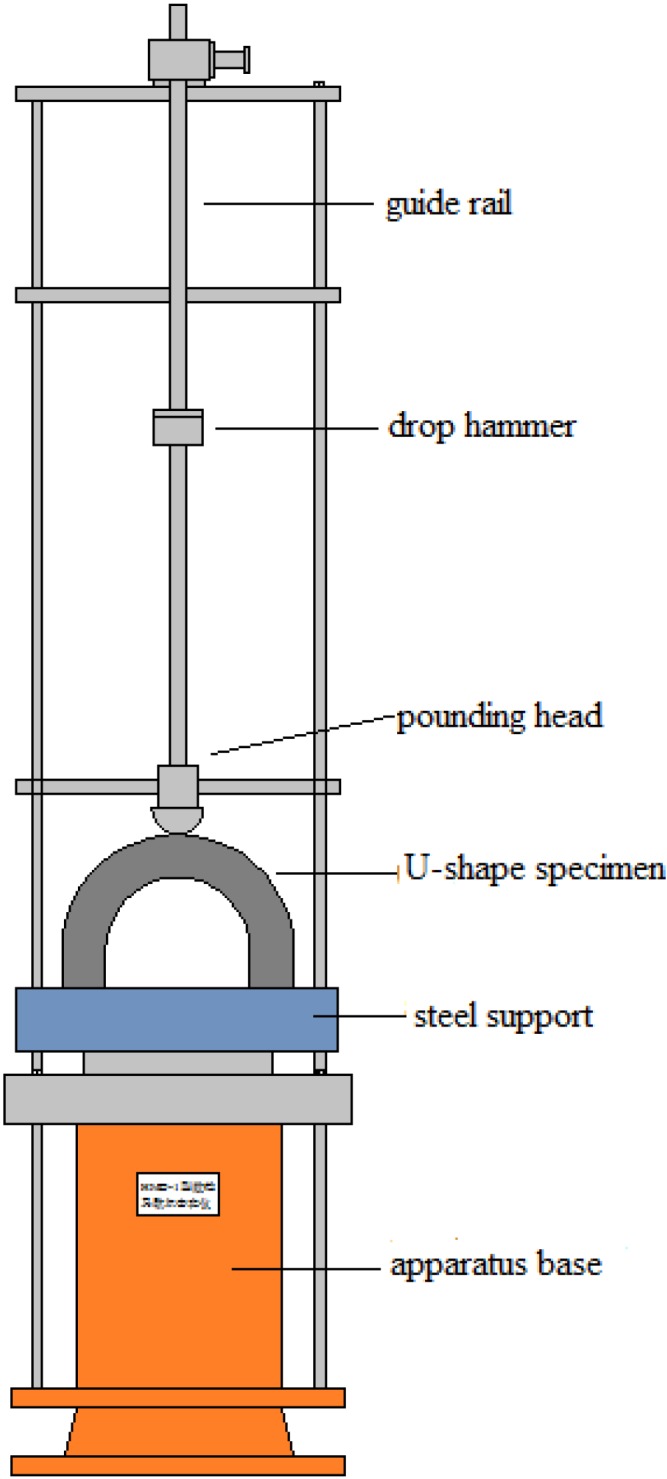
Diagram of drop-weight impact test apparatus.

The testing apparatus, illustrated in Figure 3, consists of three main components, a base, drop-weight guide system and drop hammers. Four different masses of steel hammers were used in this study, 0.875, 0.8, 0.675 and 0.5 kg, respectively. The drop-height was 400 mm. A very small gap was left between the guide rail and drop-hammer. Acetone or alcohol was used to clean the guide rail and drop-hammer, in order to minimize the friction between them. The U-shape specimen was fixed in two square grooves of the steel support. The drop-weight impact tests reported here were carried out by using a hammer from the same height of 400 mm repeatedly on a hardened steel pounding head with a hemispherical surface, which is placed on the top of the middle of U-shape specimen as shown in Figure 3.

In all tests, the same mass was dropped repeatedly from the same height until final failure occurred. For each U-shape specimen, two values should be recorded corresponding to initial and ultimate failure. The former value measures the number of blows required to initiate a visible crack, whereas the latter measures the number of blows required to ultimate failure, which should be declared when the specimen is separated into halves.

Forty-eight U-shape specimens (12 × 4 batches) were hoped to be tested in this test at first. However, a larger number of specimens—13, 12, 14 and 14 specimens from the four batches, respectively—had to be tested to achieve 12 acceptable results from each batch. Five results were rejected from four batches, in all, as they failed by cracking away from the predefined cracking path, which was shown and elaborated in the part 3 of this paper.

#### 2.3.2. Compressive Strength Test

Compressive strength of the concrete cubes was determined as per the Chinese Code (GB/T 50081-2002 [23]). The tests were carried out using a NYL-2000D hydraulic press of a 2000 kN capacity (Wuxi Jianyi Instrument & Machinery Co., Ltd., Wuxi, China) and performed on three 100-mm cubic specimens for each batch. A total of 12 cubic specimens (3 × 4 batches) were tested at the age of 28 days, as a means of quality control.

## 3. Results and Discussions

### 3.1. Compressive Strength Results

The compressive strength was calculated according to the Chinese Code (GB/T 50081-2002 [23]). A summary of statistical parameters for the results of the compression tests, carried out at the age of 28 days, is presented in Table 3. The overall average 28-day compressive strength was 44.3 MPa and the overall standard deviation was 3.27 MPa. The overall coefficient of variation was 7.38%.

**Table 3:** Compressive strength test results of concrete.

Specimen Number	Compressive Strength (MPa)
1	45.9
2	43.7
3	38.3
4	41.3
5	43.9
6	48.2
7	46.9
8	45.6
9	44.4
10	42.5
11	40.8
12	49.8
Mean (*x*)	44.3
SD (σ)	3.27
COV (σ/*x*)%	7.38

SD = standard deviation; COV = coefficient of variation.

**Figure 4:**
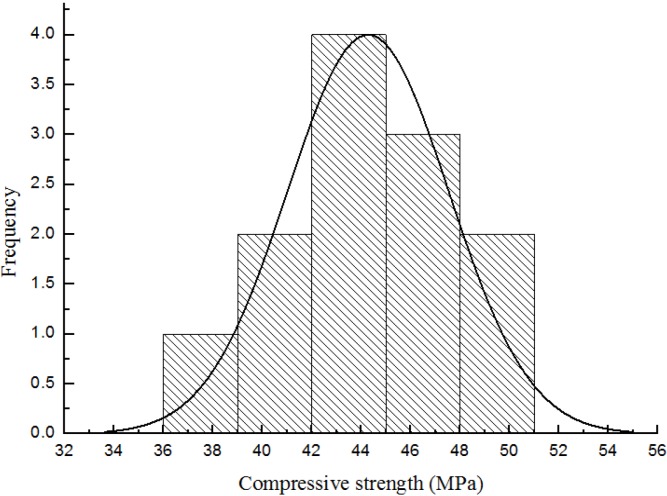
Distribution of compressive strength test results.

The origin was used in the study. Figure 4 presents the frequency histogram of the 12 results obtained from the compressive strength tests. The figure shows that the results are almost normally distributed and fit well with the superimposed normal distribution curve of the same mean and standard deviations as the compressive strength results.

The overall standard deviation indicated good quality control for the production of the concrete specimens. A value of 4–6 MPa is considered acceptable in the UK [24]. The value of the coefficient of variation shows further evidence of good quality control. The overall coefficient of variation (7.38%) is much lower than the limit of 15% suggested by Swamy and Stavrides [25] for good quality control. In addition, Day [24] suggested that a coefficient of variation between 5% and 10% generally represents a reasonable quality control.

### 3.2. Drop-Weight Impact Test Results

**Figure 5:**
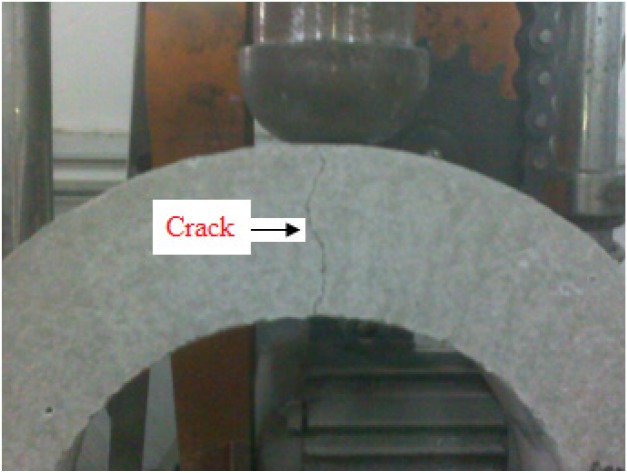
Crack occurred at the middle of U-shape specimen.

**Figure 6:**
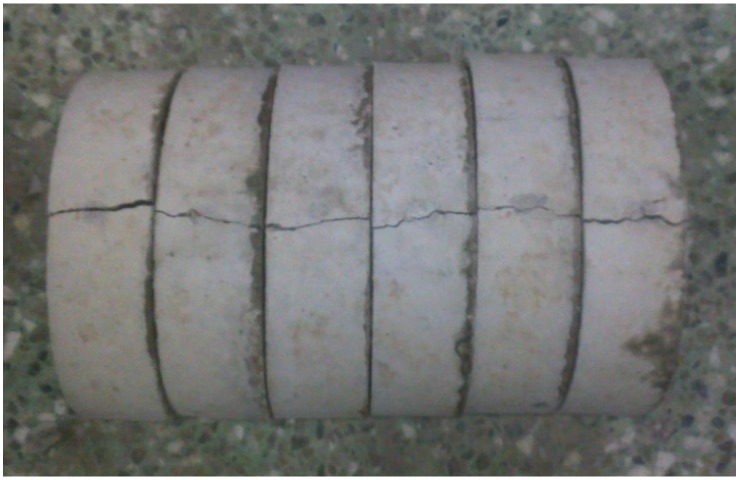
Accepted failure of U-shape specimens concrete.

As expected, the crack occurred in a predefined path, as shown in Figure 5. The initiation of crack and the final fracture occurred at the bottom and top surfaces in the middle of the U-shape specimen, and a single crack split the specimen in two, as shown in Figure 6. In future work, it is suggested that the hammer mass and/or the drop-weight should be adjusted, so that at least 10 blows are required to fail the test specimen, in order to facilitate observing the crack initiation and propagation.

The first-crack (*N*_1_) and the ultimate failure (*N*_2_) impact resistance values obtained from the drop-weight impact test are given in Table 4. The statistical parameters are also presented in Table 4 for all results. The standard deviation values obtained in this study are high, and the range of standard deviation is wide. This is also reported in the literature. However, unlike compressive strength, it is not realistic to use the standard deviation to judge or compare the impact resistance results. This is because the impact test is not a standard test. In such cases, it is more appropriate to use the coefficient of variation [12]. The coefficient of variation is considered a more meaningful index of variability because it accounts for the mean as well as the standard deviation. Day [24] stated that several ACI committees including 212 (mixture proportioning), 214 (evaluation of test results) and 363 (high strength concrete) have adopted the coefficient of variation as a measure of variability rather than the standard deviation. The maximum of coefficient of variation in this study is 31.2%. It is lower than the values obtained from the ACI impact test in the literatures. This can be easily attributed to the U-shape specimens used in this study, which can predetermine the location of the crack and reduce the scatter of the results.

**Figure 7:**
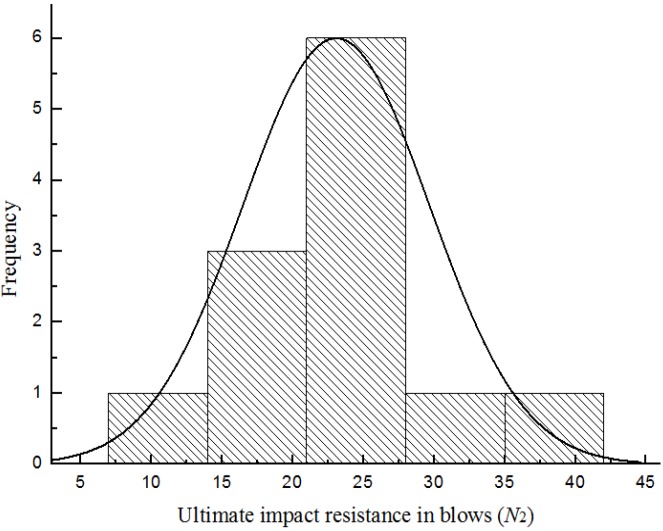
Distribution of the ultimate impact resistance for batch 1 (0.875 kg).

**Figure 8:**
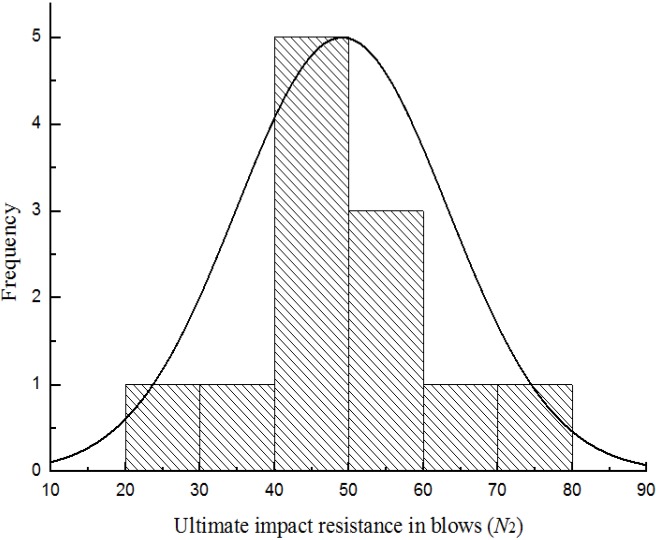
Distribution of the ultimate impact resistance for batch 2 (0.8 kg).

**Figure 9:**
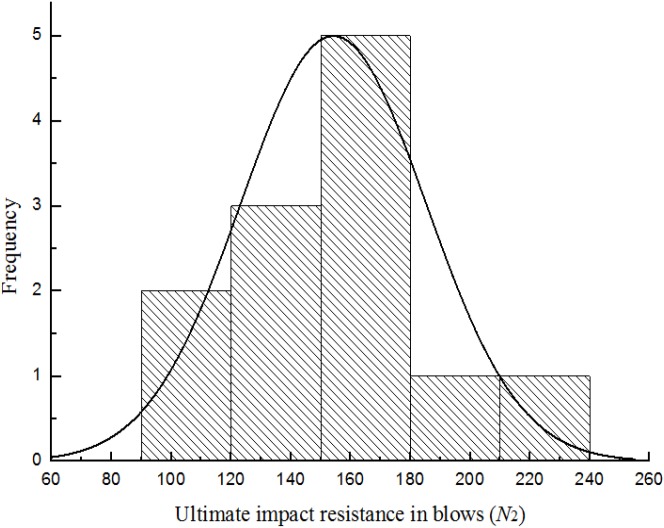
Distribution of the ultimate impact resistance for batch 3 (0.675 kg).

**Figure 10:**
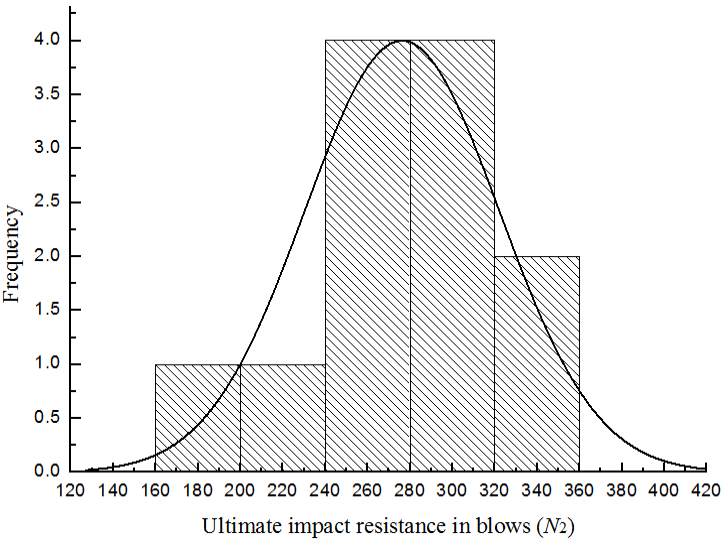
Distribution of the ultimate impact resistance for batch 4 (0.5 kg).

Figure 7, Figure 8, Figure 9 and Figure 10 show the frequency histogram of the ultimate failure impact resistance results with the normal distribution curve overlapping them for all test specimens, respectively. It can be seen that the distributions of the results have departed from the normal distribution. The departure from the normal distribution gives further evidence of scatter in the impact resistance results. Again, similar conclusions can be drawn for the case of first-crack impact resistance results but charts are not presented here. This indicated that the impact resistance results are poor to follow a normal distribution.

**Table 4:** Impact resistance test results for U-shape specimens (blows).

Specimen Number	0.875 kg		0.8 kg		0.675 kg		0.5 kg	
	*N*_1_	*N*_2_	*N*_1_	*N*_2_	*N*_1_	*N*_2_	*N*_1_	*N*_2_
1	16	24	38	45	138	163	236	269
2	18	27	49	56	160	173	251	277
3	8	12	23	28	85	106	182	218
4	20	25	60	68	121	130	293	338
5	17	23	31	40	136	146	308	347
6	19	28	36	42	172	189	169	185
7	18	26	43	52	196	213	269	296
8	15	20	51	56	117	134	257	283
9	9	15	31	40	96	112	229	251
10	16	23	29	36	136	156	257	289
11	25	37	65	79	134	158	223	256
12	12	17	40	47	156	172	285	306
Mean (*x*)	16	23	41	49	137	154	247	276
SD (σ)	4.7	6.6	12.8	14.2	31.1	31	41.9	45.8
COV (σ/*x*)%	29.4	28.7	31.2	29.0	22.7	20.1	17.0	16.6

SD = standard deviation; COV = coefficient of variation. *N*_1_ = first-crack impact resistance in blows; *N*_2_ = ultimate failure impact resistance in blows.

### 3.3. Regression Analysis

**Figure 11:**
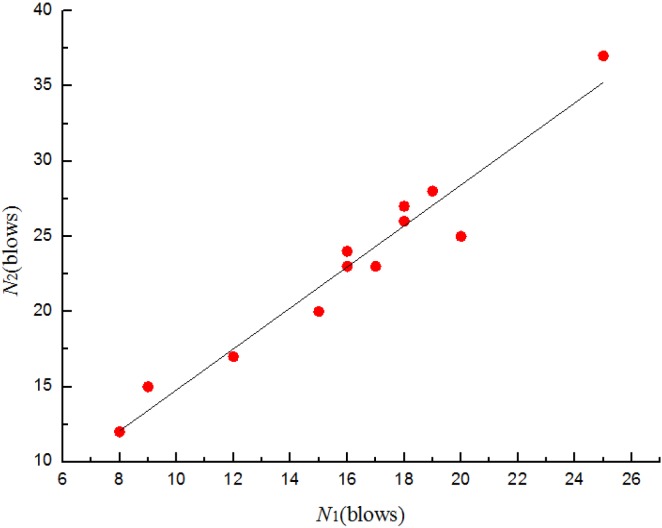
Scatter diagram of impact data with fitted regression line for batch 1 (0.875 kg).

**Figure 12:**
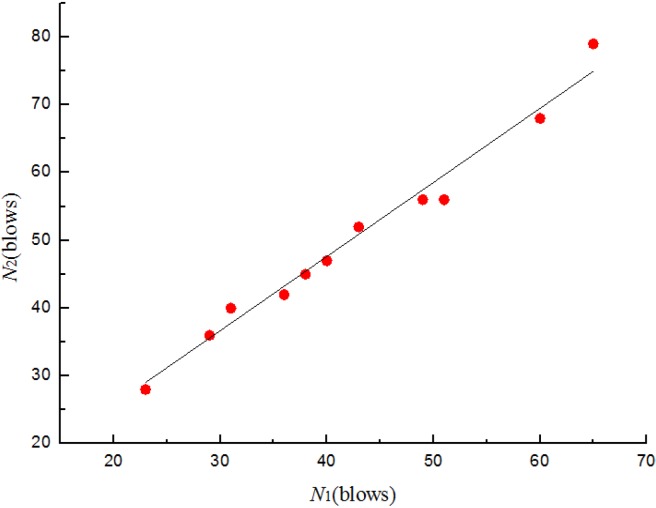
Scatter diagram of impact data with fitted regression line for batch 2 (0.8 kg).

**Figure 13:**
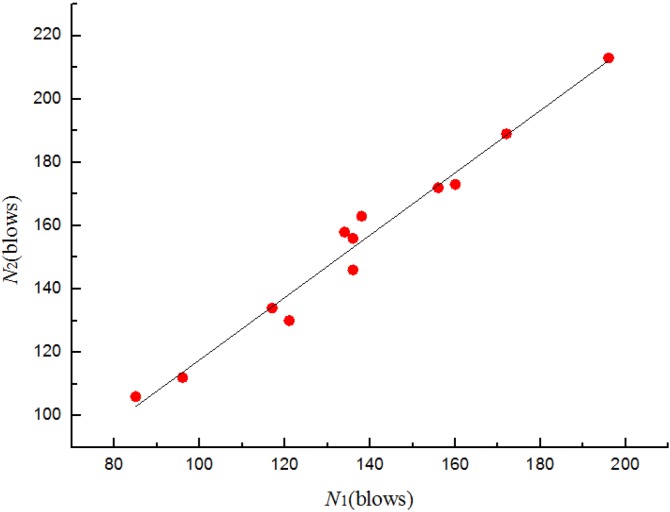
Scatter diagram of impact data with fitted regression line for batch 3 (0.675 kg).

**Figure 14:**
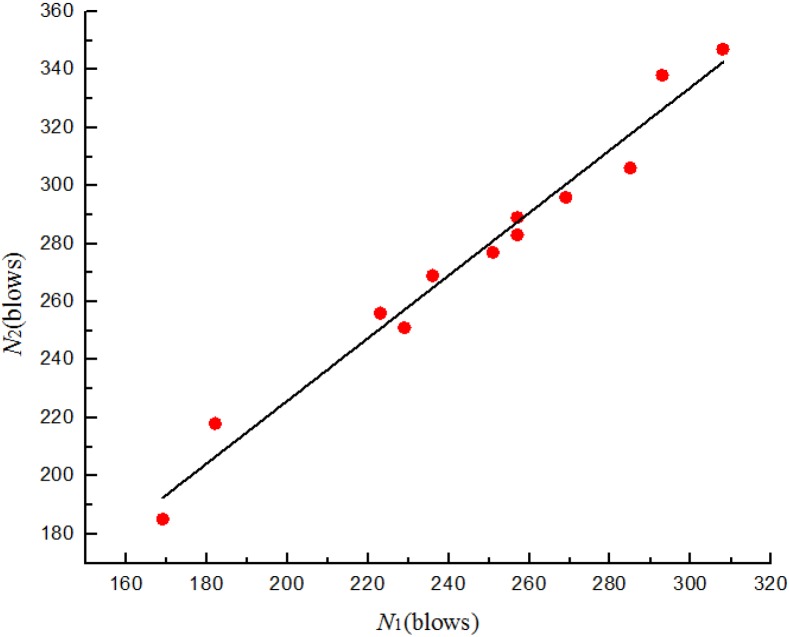
Scatter diagram of impact data with fitted regression line for batch 4 (0.5 kg).

As per the theory of linear regression analysis, the positive linear relationship between the first-crack and ultimate failure impact resistance is described using the following simple linear regression model:

(1) $$N_{2}=a+b\times N_{1}$$

where *N*_2_ is the ultimate failure impact resistance in blows and *N*_1_ is the corresponding first-crack impact resistance in blows, *a* and *b* are unknown constants. Substituting into Equation (1) and applying to the least square method, the relationships between *N*_1_ and *N*_2_ for four batches specimens are shown from Figure 11, Figure 12, Figure 13 and Figure 14. The parameter values of the linear regression analysis are presented in Table 5.

**Table 5:** Parameter values of the linear regression analysis.

Mass of Drop Hammer	*a*	*b*	*R*^2^
0.875 kg	1.156	1.363	0.941
0.8 kg	3.884	1.094	0.976
0.675 kg	19.136	0.985	0.972
0.5 kg	10.073	1.079	0.97

*R*^2^ = coefficient of determination.

Based on the regression analysis of the impact resistance results (Figure 11, Figure 12, Figure 13 and Figure 14 and Table 5), the linear relationship between *N*_1_ and *N*_2_ of four batches specimens were supported by the values of 0.941, 0.976, 0.972 and 0.97, respectively, for the coefficient of determination (*R*^2^). The prediction equations developed for the ultimate failure impact resistance in blows were as follows:

(2) $${\hat{N}}_{2}=\;1.156\;+\;1.363N_{1}\;\mathrm{for}\;\mathrm{batch}\;1\;(0.875\;\mathrm{kg})$$

(3) $${\hat{N}}_{2}=\;3.884\;+\;1.094N_{1}\;\mathrm{for}\;\mathrm{batch}\;2\;(0.8\;\mathrm{kg})$$

(4) $${\hat{N}}_{2}=\;19.136\;+\;0.985N_{1}\;\mathrm{for}\;\mathrm{batch}\;3\;(0.675\;\mathrm{kg})$$

(5) $${\hat{N}}_{2}=10.073+\;1.079N_{1}\;\mathrm{for}\;\mathrm{batch}\;4\;(0.5\;\mathrm{kg})$$

where ${\hat{N}}_{2}$ is the ultimate failure impact resistance in blows corresponding to *N*_1_ as the experimentally measured first-crack impact resistance in blows. Most statisticians consider a coefficient of determination of 0.7 or higher for a reasonable model [26]. Therefore, the derived equations may successfully be used to represent the relationship between *N*_1_ and *N*_2_ of U-shape specimens.

### 3.4. Minimum Number of Specimens

In probability and statistics, Student’s *t*-distribution is more appropriate to estimate the population mean *in situ* ations where the sample size is small and population standard deviation is unknown. Therefore, the theory of Student’s *t*-distribution was used in this study. The coefficient of variation presented in Table 4 has another valuable practical application. Swamy and Stavridis [25] showed that it can be used to determine the minimum number of tests, *n*, required in order to guarantee that the percentage error in the measured average value (that is the percentage error between sample mean and population mean) is below a specified limit, *i.e.*, at a specific level of confidence, as given by Equation (6) below.

(6) $$n=\;t^{2}v^{2}/e^{2}$$

where *v* is the coefficient of variation; *t* is the value of Student’s *t*-distribution for the specified level of confidence and is dependent on the degree of freedom, which is related to the number of tests. If the number of tests is *n*, then the Student’s *t*-distribution has *n* − 1 degrees of freedom.

For a large sample size, *t* approaches 1.645 and 1.282 at 95% and 90% levels of confidence, respectively [27,28]. Table 6 presents the number of specimens required to keep the error under various limits between 5% and 30%, at the 95% and 90% levels of confidence. It can be seen that for the U-shape specimen, if the number of tests is 12 at the 95% and 90% levels of confidence, the error in the measured average value is to be kept below 15%; and if the error is to be kept under 10%, the minimum number of specimens should be 23 and 14 at the 95% and 90% levels of confidence. Moreover, if four specimens are tested to determine the impact resistance and the average is reported, then the error in average measured value could be between 20% and 25% depending on the level of confidence, as shown in Table 6.

**Table 6:** Number of specimens required to keep the error under a specific limit.

Level of Confidence	Error (*e*%)	0.875 Kg		0.8 Kg		0.675 Kg		0.5 Kg	
		*N*_1_	*N*_2_	*N*_1_	*N*_2_	*N*_1_	*N*_2_	*N*_1_	*N*_2_
95%	<5	93	89	104	90	56	44	31	30
	<10	23	22	26	23	14	11	8	7
	<15	10	10	12	10	6	5	4	3
	<20	6	6	7	6	4	3	2	2
	<25	4	4	4	3	2	2	1	1
	<30	3	3	3	3	2	1	1	1
90%	<5	56	54	63	55	34	27	19	18
	<10	14	13	16	14	9	7	5	5
	<15	6	6	7	6	4	3	2	2
	<20	4	3	4	3	2	2	1	1
	<25	2	2	3	2	1	1	1	1
	<30	2	2	2	2	1	1	1	1

## 4. Conclusions

In this paper, experiments and statistical analysis were performed to study concrete impact resistance. Tests were carried out using a newly designed drop-weight impact test apparatus, and the following conclusions from this research can be drawn:

- Using U-shape specimens can force cracks to occur in a predefined path, and for each specimen, just a single crack occurred, which can facilitate observing the crack initiation and propagation. The newly designed drop-weight impact test apparatus is feasible and the operation is easy. It is also simple and intuitive to evaluate and compare the influence of different types of fibre reinforcement on the impact strength of concrete.
- The drop-weight impact test results showed good reproducibility with coefficients of variation in the range of 15%–30%. It was due partly to the use of U-shape specimens which predetermined the location of the crack.
- Four linear models have been suggested for four different impacting masses. In addition, the minimum number of specimens should be 23 and 14 at the 95% and 90% levels of confidence, in order to keep the error below 10%.

Current understanding of the impact resistance of concrete is limited. At the heart of the problem is the absence of a standardized test technique for testing concrete under impact load. Therefore, much still remains to be done both towards the development of a standardized technique and towards developing a comprehensive understanding of concrete performance under impact load.
